# Web based tools for visualizing imaging data and development of XNATView, a zero footprint image viewer

**DOI:** 10.3389/fninf.2014.00053

**Published:** 2014-05-27

**Authors:** David A. Gutman, William D. Dunn, Jake Cobb, Richard M. Stoner, Jayashree Kalpathy-Cramer, Bradley Erickson

**Affiliations:** ^1^Department of Biomedical Informatics, Emory UniversityAtlanta, GA, USA; ^2^Georgia Institute of Technology, College of ComputingAtlanta, GA, USA; ^3^Department of Neurosciences, University of California San Diego School of MedicineLa Jolla, CA, USA; ^4^Harvard-MIT Division of Health Sciences and Technology, Martinos Center for Biomedical ImagingCharlestown, MA, USA; ^5^Department of Radiology, Mayo ClinicRochester, MN, USA

**Keywords:** radiology, MRI, DICOM-viewer, XNAT, web-based image viewer, PyXNAT, biomedical imaging

## Abstract

Advances in web technologies now allow direct visualization of imaging data sets without necessitating the download of large file sets or the installation of software. This allows centralization of file storage and facilitates image review and analysis. XNATView is a light framework recently developed in our lab to visualize DICOM images stored in The Extensible Neuroimaging Archive Toolkit (XNAT). It consists of a PyXNAT-based framework to wrap around the REST application programming interface (API) and query the data in XNAT. XNATView was developed to simplify quality assurance, help organize imaging data, and facilitate data sharing for intra- and inter-laboratory collaborations. Its zero-footprint design allows the user to connect to XNAT from a web browser, navigate through projects, experiments, and subjects, and view DICOM images with accompanying metadata all within a single viewing instance.

## Introduction

Data management challenges regularly pose problems among imaging laboratories. Visualization and sharing of complex imaging data sets has traditionally involved downloading large file sets, installing custom software applications, or in some cases simply sharing screenshots of specific images with colleagues for review and comment. These inefficient *ad hoc* solutions often make collaboration and soliciting feedback on imaging data complicated, imprecise, and time intensive. However, recent advances in web-based technologies such as HTML5 and faster overall internet connectivity have the potential to significantly simplify this process.

In this work, we review some of the existing source tools and libraries that facilitate web-based image visualization of imaging data sets. While DICOM is the *lingua franca* of clinical imaging, it is worth noting many other imaging formats are commonly used in imaging research. We will therefore also review some tools and frameworks that support imaging formats in addition to DICOM.

A number of emerging technologies allow visualization and interaction with not only the image itself, but also with derivative images such as masks, tractography results, and statistical maps. We will review various tools currently available that support this functionality offline as well as demonstrate our current work that allows visualization of image overlays directly via HTML.

Capitalizing on these recent advances, we have developed a light weight HTML based image browser that integrates XNAT, a popular research informatics platform which we will describe later. The need for such an image-viewer stemmed from an ongoing project in our lab which involved the organization and curation of large retrospectively-collected imaging data sets of cancer patients with high grade gliomas. For our initial project (Gutman et al., 2013), we were presented with hundreds of volumes of MR imaging sets and we needed an efficient method to select which MRI cases were appropriate for the study. Specifically, we wanted to pull patients with pre-surgical/pre-treatment T2 FLAIR as well as both pre and post gadolinium contrast T1 sequences with sufficient image quality. Unlike typical neuroimaging studies where data is collected on a single MRI machine with a well-defined imaging protocol, the imaging data in this case was collected during a period of more than a decade from various universities within the TCGA network (Cancer Genome Atlas Research et al., 2013), oftentimes using different image scanners and following different protocols.

Due to the heterogeneity of imaging protocols in the clinical setting, our data was interspersed with DICOM images of insufficient quality (motion artifacts, limited field of view, missing slices) as well as ambiguous/improper names (i.e., we had found that several T1 images had been incorrectly labeled as T2). This task was further confounded by the necessities of anonymization, as well as by a lack of a direct link back to clinical data that would have indicated the treatment status of the patient at a given scan (e.g., pre/post-surgery). Additional complications included numerous seemingly duplicate/extraneous scans (e.g., two consecutive images labeled “T2 FLAIR,” or three different images all labeled “T1 Gad”) which needed to be disambiguated. In a clinical environment, a scan technician may repeat a scan due to poor image quality without needing a way to label the “good” scan. As this data is of course not available many years later when the data is being analyzed for research purposes, the ability to rapidly open and compare images is thus critical. Due to the massive volume of patient data, we therefore wanted some type of quality assurance tool that we could use to browse through the images to quickly determine if the corresponding labels and metadata were correct.

While a built-in image viewer is available through XNAT, the time required to select and to view individual scans from patient to patient made this option too inefficient for our project. In addition, the viewer uses Java, which presented a number of practical challenges, where programs can not readily be updated (e.g., Java) on university- or hospital-owned equipment. To address these issues, we developed XNATView, a tool that allows us and potentially any lab involved in large population neuroradiological research to easily review large sets of image sequences solely from a web browser.

While currently supporting direct integration with XNAT, the XNATView interface can be easily modified to communicate with any service that supports query and retrieval of DICOM images. As a proof of principle, we will present some of our prototype work integrating XNATView, through various plugins, with the Platform to Enable Shared Scientific Computing And Research Advances (PESSCARA) being developed at the Mayo Clinic. We have used the term zero-foot print viewer to describe software that does not require installation of additional software (e.g., Java, Active-X, Flash, etc.) relying on native functionality of the web browser.

## Background

### Research image management systems/PACS

The basic technology to support the standardized sharing of medical images is a Picture Archiving and Communication System (PACS) (Bryan et al., 1999). The basic structure includes a secure one-way interface transmitting DICOM formatted images from the physical data capture (X-ray, CT, MRI, etc.), which are ideally (although optionally) transmitted to a quality assurance workstation (PACS gateway) where demographics and other characteristics are verified. The images are then transferred to a centralized archive, where they can be queried and viewed by radiologists in a reading workstation. Numerous vendors have developed their own PACS workstations, with varying capabilities ranging from simply browsing 2-D slices to allowing 3-D visualizations and advanced image reconstruction capabilities.

For the purposes of this review, we will limit ourselves to freely-available open-source based platforms. Resources such as http://idoimaging.com and http://nitrc.org list a number of available medical software packages as well as accompanying information of the tools.

**Figure 1:**
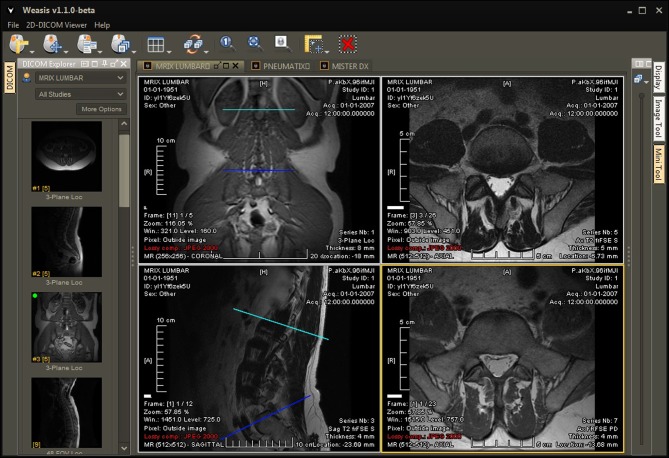
**User interface of Weasis, a program available through the DCM4CHE imaging collection**. Weasis offers several tools to facilitate viewing large amounts of clinical data, such as line drawing, measurements, and a magnifying window. For more screenshots, visit http://www.dcm4che.org/confluence/display/WEA/Home.

Among the most versatile and earliest implementations of an open-source web-based viewer is Weasis, a program available through the DCM4CHE application collection. DCM4CHE is a DICOM archive and image manager that can be entirely run from a web browser (http://dcm4che.org/). It was developed within the framework of JDicom, a toolkit written in Java (Warnock et al., 2007) and is currently distributed by the developers of the DCMTK toolkit (DICOM@OFFIS, 2013). Personal correspondences have highlighted that DCM4CHE is especially appropriate for large databases, such as those on a university or hospital setting, and runs smoothly on Windows or Linux machines. Importantly, DCM4CHE offers various storage, clinical, and sharing features which were designed around contemporary standards such as HL7 and DICOM to facilitate interoperability between users. DCM4CHE can serve as an image source for DICOM compliant applications (such as ClearCanvas and OsiriX/etc.) as well as an integrated web-based image viewer through the Weasis application. Weasis is a multipurpose clinical image viewer designed to view images stored in a PACS with minor adjustments (Figure 1). CDMedicPACSWeb provides (http://cdmedicpacsweb.sourceforge.net/CDMEDIC_PACS_WEB.html) a virtual machine/base installation which has WEASIS and DCM4CHEE preconfigured.

While these tools are useful when DICOM is the primary imaging modality, other more comprehensive systems that support multiple imaging formats have also been developed.

A popular PACS workstation program on the Macintosh Platform is OsiriX (OSIRIX, 2004), which has both a free open-source version as well as a more fully featured and FDA-approved version (which includes a certified PACS-viewer) which is appropriate if the tool is to be used for diagnostic purposes (Rosset et al., 2004). ClearCanvas Workstation (ClearCanvas Ontario, CA http://clearcanvas.ca) provides similar functionality for the Windows environment and also features both a paid FDA-approved version as well as a free open-source version which has been demonstrated to facilitate inter-rater agreement (Hsieh et al., 2013). ClearCanvas also supports plugins, making it adaptive to specific user needs. For example, our lab (Gutman et al., 2013) previously used a plugin that permits the use of the AIM markup language (Channin et al., 2009) for structured annotations. In addition to standard editions, ClearCanvas also offered a beta-release version that supported an integrated Web Viewer, although the current status of that project is unclear.

Another useful open-source project is the Medical Imaging Interaction Toolkit (MITK) which offers the user data management, advanced visualization, and interactive functions (http://www.mitk.org/MITK). The basic framework offers advantages of both Insight Toolkit (ITK) and Visualization Toolkit (VTK) and supports a wide variety of application plugins (some open-sourced, others not) to customize the user experience. For example, the “Iso Surface” plugin interpolates user-defined pixel selections and creates surface structures on regions of interest and the “IGT Tracking” plugin allows one to connect a tracking device to the image and record the resulting tracking location data. One aspect worth highlighting is the MITK Diffusion Imaging component which offers a suite of visualizations including fiber tractography, Q-Ball reconstruction, and Fiberfox to generate complex white matter tissue models.

InVesalius, a Brazilian program now in its third version, is another convenient tool used to view DICOM files from both CT and MRI protocols. It offers wide versatility and can be run on MS Windows, GNU Linux, and soon MacOS X systems. One of its main features is its detailed image reconstruction capability (http://svn.softwarepublico.gov.br/trac/invesalius/wiki/InVesalius/Screenshots).

Other lighter weight image viewers exist as well, such as the NIH ImageJ program (http://rsb.info.nih.gov/ij/), which supports DICOM (among many other formats), and IrfanView (http://www.irfanview.com/).

### COINS, MIDAS, PESSCARA, XNAT: research informatics platforms

Public imaging informatics systems are designed to complement the limitations of a clinically-focused PACS and allow for the smooth exchange of data between investigators to facilitate research. They also generally support other image formats besides DICOM that are commonly used as intermediates during image analysis.

#### COINS—collaborative informatics and neuroimaging suite

**Figure 2:**
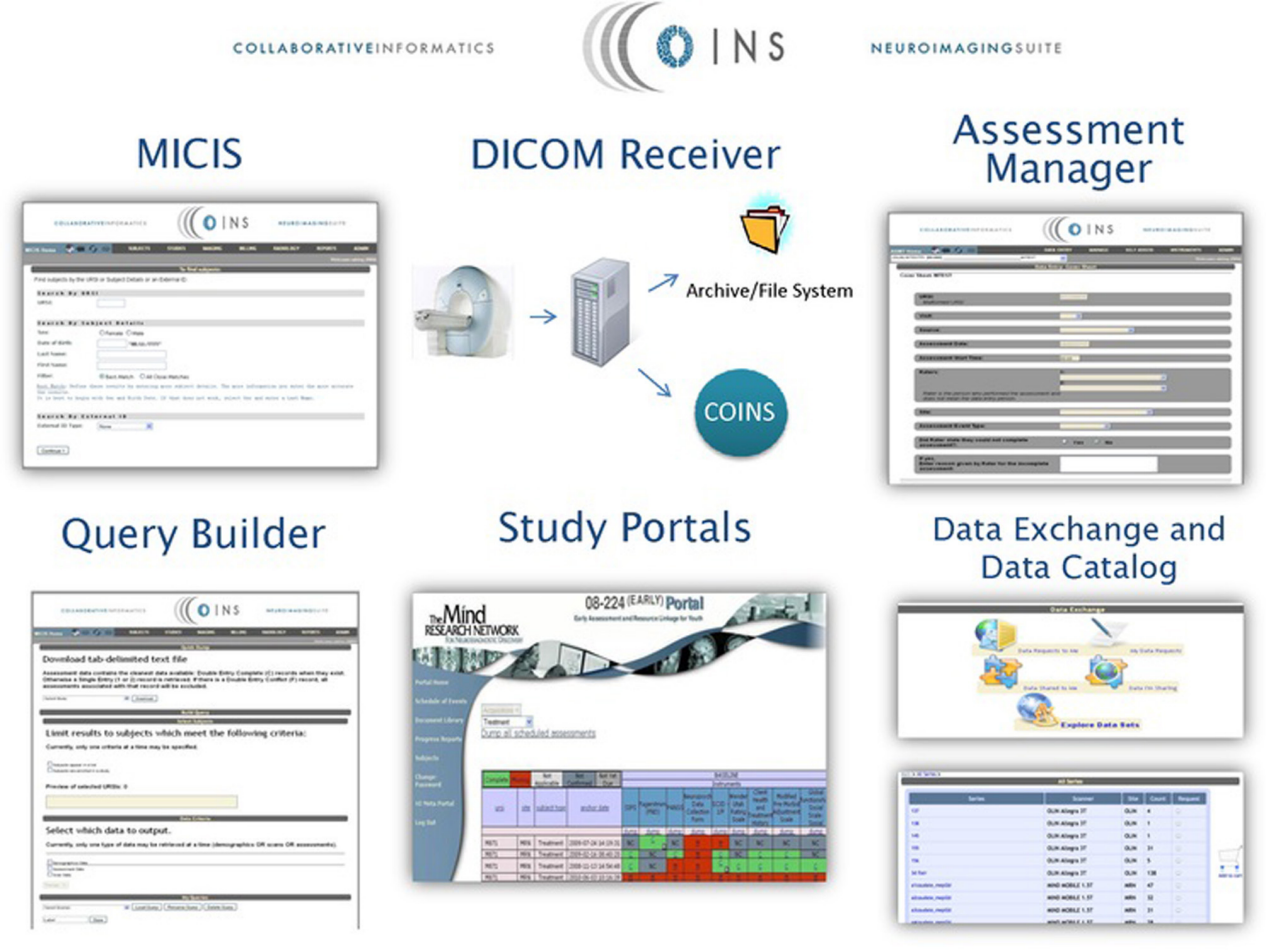
**An overview of the COINS web-based neuroimaging software suite**. “COINS Tools: MICIS—Participant enrollment and management, MRI imaging data import, Scan annotation and behavioral data management, Radiology review event reports, Scan time billing. DICOM Receiver—Automates image archiving to file system and storage of meta-data to MICIS. Assessment Manager—Single and double entry as well as self-assessment. Query Builder—Secure, *ad-hoc* querying of single and cross-site studies for assessments, scans and demographics. Study Portals—Progress reports for subject tracking, shareable documents (study measures, meeting notes, etc.). Data Exchange with Data Catalog—Browse, request and share data, available for imaging data and clinical assessments, tracks data requests and keeps an inventory of data.” (http://neuroinformatics2012.org/abstracts/coins-collaborative-informatics-neuroimaging-suite-give-get-collect).

The COllaborative Informatics and Neuroimaging Suite (COINS) was developed at the Mind Research Network headquartered in Albuquerque, New Mexico and currently holds imaging data from more than 20,000 participants (Scott et al., 2011). COINS is an online portal where imaging data, as well as reports, annotations, and billing data, can be automatically archived into the system via a DICOM receiver. Additional accompanying data from interviews, questionnaires, and neuropsychological tests can also be entered through a web application called Assessment Manager (ASMT, Figure 2). COINS also features a Data Exchange Tool designed to facilitate communication by allowing de-identified neuroimaging datasets with associated metadata to be shared between collaborating research groups. In addition, a Medical Imaging Computer Information System (MICIS) component allows smooth project creation and participant enrollment and management, making COINS a useful tool in human research and clinical studies. In addition to overcoming complicated challenges involved with human subjects and PHI, principal investigators are able to set permissions assigning different levels of access according to various guidelines set by the Institutional Review Board.

#### MIDAS—the multimedia digital archiving system

**Figure 3:**
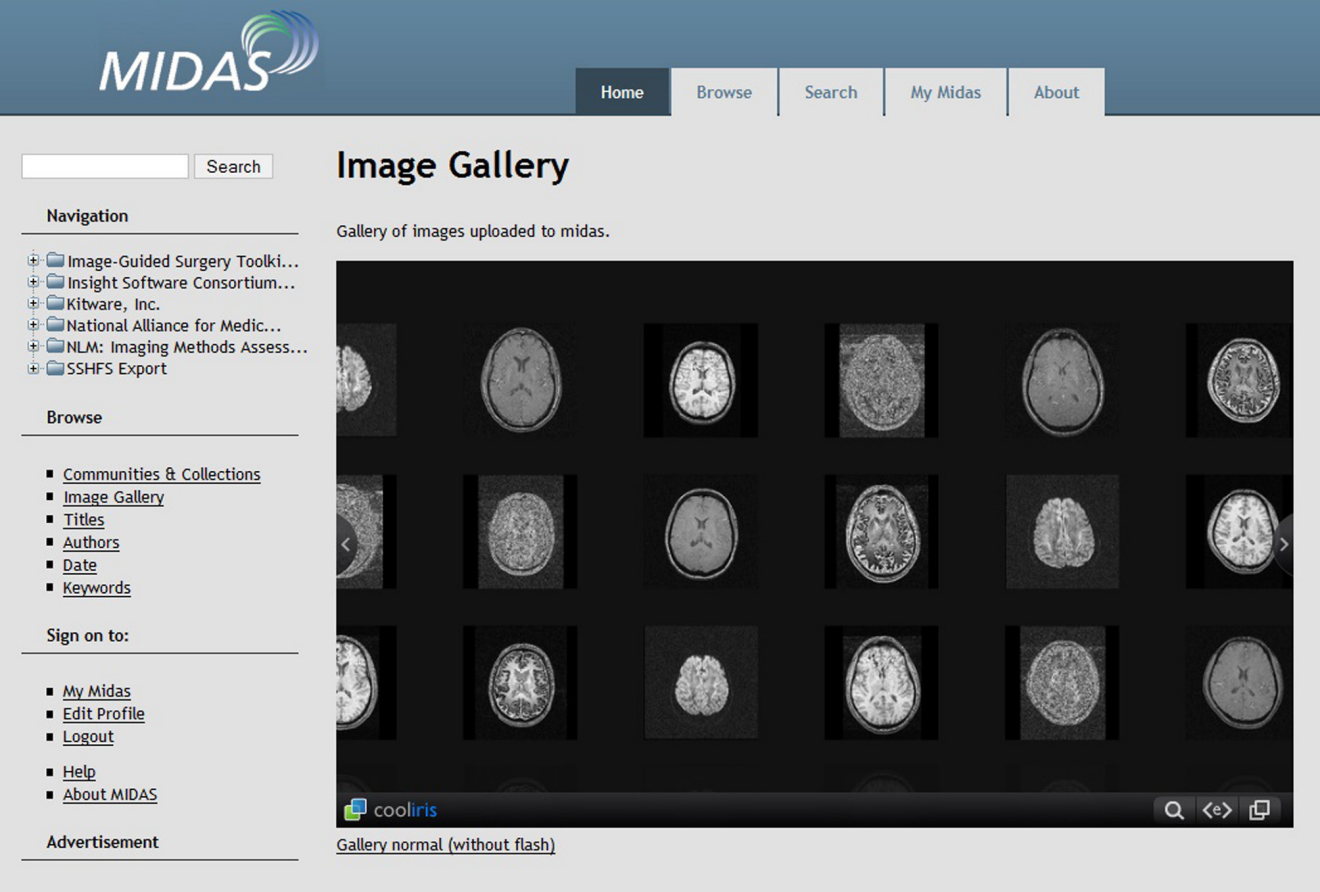
**Screenshot of the Midas Platform**. Midas is a web-based toolkit that allows facilitated review of clinical imaging data through digital storage, online reporting, interactive visualization, and server-slide processing (http://www.midasplatform.org/MIDAS/resources/toolbox.html).

The Midas Platform is a PHP-based data storage system designed to facilitate computational scientific research by integrating data from a variety of sources (http://www.midasplatform.org/) (Figure 3). MIDAS was developed and is maintained by the same developers behind the VTK toolkit, which is commonly used throughout many imaging analysis modalities. The open source software, now in its 3.2.8 version, indexes data sources from imaging databases and visualization tools. The Midas framework can then query the back-end database. One benefit of the platform is its ability to be highly customized with various plugins to individually tailor the program to specific research needs.

#### PESSCARA—platform to enable shared scientific computing and research advances

PESSCARA is a platform based on open-source resources and combines four important components for the conduct of science. The first components are image data and metadata stores, obviously critical starting points. We use the open source DCM4CHEE software to provide the mechanism for receiving and sending DICOM data. In most cases, there are processing steps applied to the medical images, including filtering, registration, segmentation, etc. These steps create new versions of the images, or add metadata about the images. Content management systems were built to do exactly these functions, and so we have leveraged the TACTIC CMS as the second major component of PESSCARA. It is open-source and has a Python application programming interface (API) to allow automation of many steps.

The third component of PESSCARA is an algorithm development environment. For that, we selected iPython Notebooks. Python has become the major programming language of science because of powerful libraries that can efficiently handle most tasks, because the language itself is easy to understand and has free interpreters for all major operating systems, and because the iPython Notebook provides a flexible way to develop, share, and document algorithms. Python has powerful image processing libraries, and also powerful data analysis tools. A mechanism for documenting the complete processing flow, including input data, processing steps, and results is key to shareable science. The fourth and final component is a results repository that allows a user to document and share all of the parts. This can allow other investigators to validate the results, as well as to test the same processing steps on other data sets or other processing algorithms on the same data set. We are currently in the process of posting the code and a virtual machine instance of this framework (http://PESSCARA.org).

#### XNAT—the extensible neuroimaging archive toolkit

XNAT is an open source imaging informatics platform developed at Washington University in St. Louis and was designed for the storage and management of large heterogeneous imaging data sets to facilitate neuroradiological research (http://xnat.org/) (Marcus et al., 2007). The extendibility allows each research group to customize an “instance” and extend the basic application to suit their needs. Originally developed to store Phillips PAR files, the application now has a robust DICOM image management system and also allows storage of other common imaging formats (NII, Analyze, MGZ, etc). XNAT provides key functionality such as uploading and downloading data in various formats, organizing and sharing data, and customizing security and access to the data. In XNAT, users are able to save the original or modified files to disks or send them across a network to a DICOM C-STORE service class provider, such as a PACS or another XNAT instance. In addition, XNAT provides a means to view the data using a built-in Java-based DICOM viewer. The viewer relies on plugins to implement image-type-specific functionality and additional plugins can be developed and integrated to customize the viewer.

One of the key advantages of XNAT and similar systems relative to a more traditional PACS-based image management system is the flexibility provided in “tagging” data. Certain features that are critical in a research setting, such as the ability to associate certain patients with certain research protocols, are not easily handled in a typical PACS. A PACS viewer is usually organized around selecting by patient name, doctor who ordered the study, imaging modality or scan date—data that is oftentimes superfluous outside of the clinic. Once image sets are tagged in XNAT, patients can be neatly organized into projects and sorted by name, ID, or other relevant features.

### Web-based visualizations

#### The X Toolkit

Emerging technologies, including webGL and increased performance of JavaScript engines, now allow both 2D and 3D image manipulation on the client side. The X Toolkit (XTK, http://www.goXTK.com) and BrainBrowser (https://brainbrowser.cbrain.mcgill.ca/) are two popular tools that allow visualization and interaction with both 2-D (i.e., texture files .png, .jpg) and 3-D volumes, as well as the support of masks, tractography results, and/or label maps.

The X Toolkit is available on GitHub and can be used to visualize a wide spectrum of physiological phenomena ranging from white matter cortical connections, aneurysm characteristics, and knee morphologies. Of note, an interesting JavaScript library, jsdicom, also supports native DCM reading of DICOM files and is available on GitHub (https://github.com/Infogosoft/jsdicom).

An attractive feature of the XTK platform is that apart from being a native JavaScript library that directly parses DICOM files directly (as opposed to requiring a server side plugin to transcode .dcm files into .jpg/.png images), XTK can also support a number of other common neuroimaging formats such as several compressed and uncompressed formats of DICOM files (.nrrd, .nii, .nii.gz, .mgz, .dcm, etc.) as well as files from higher level MR processing (.trk, .stl, .fsm., .label, etc.) commonly used in image analysis research.

**Figure 4:**
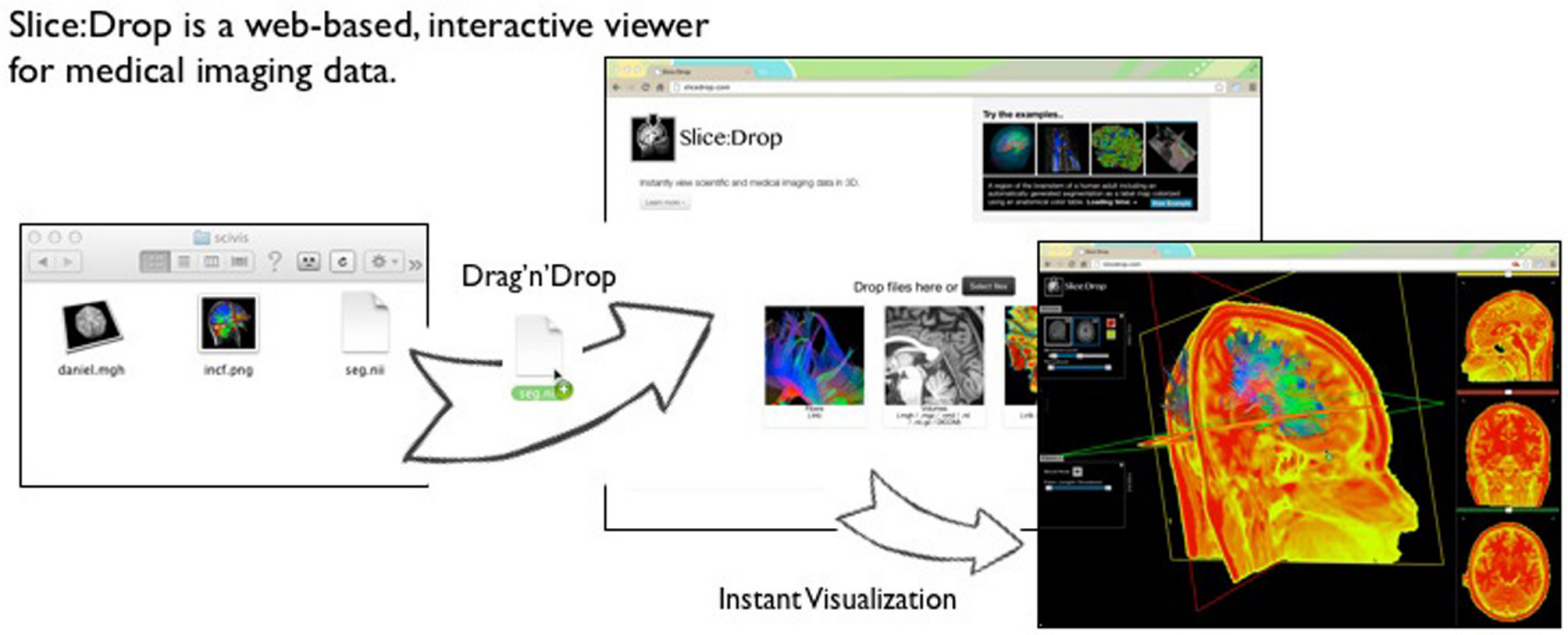
**Slice:Drop An interactive visualization tool that allows users to instantly visualize imaging data from a wide variety of compatible imaging formats (available at http://slicedrop.com/)**.

**Figure 5:**
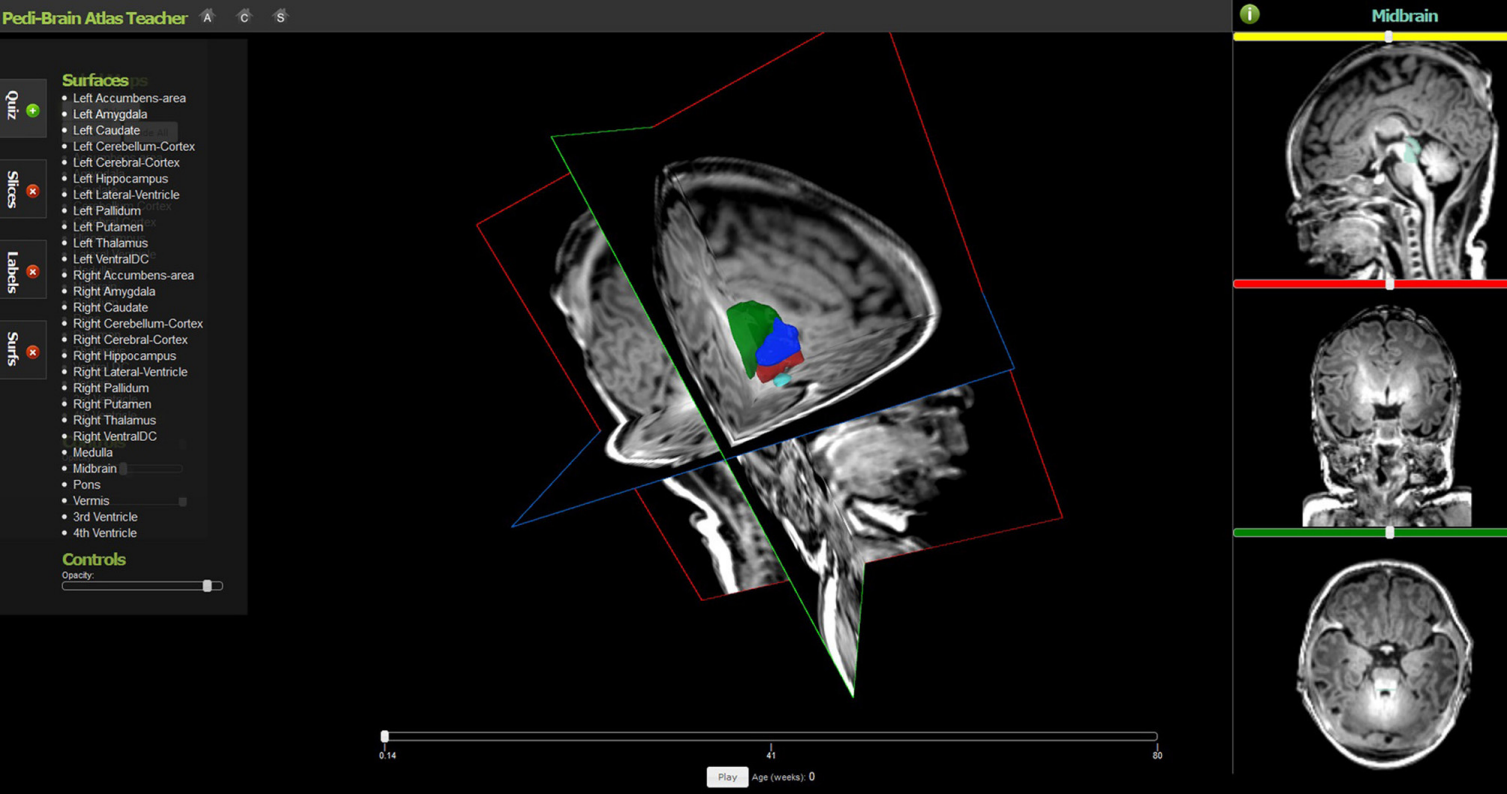
**The Pedi-Brain Atlas Teacher, an interactive visualization tool for pediatric brain tumors on MRI (available at: http://fnndsc.github.io/babybrain/)**.

Several implementations of The X Toolkit include the AneuRisk Web repository (http://mox.polimi.it/it/progetti/aneurisk/) (Ford et al., 2009) and SliceDrop.org (Figure 4). The LONI group (formerly of UCLA, now of UCSC) has developed an extension of SliceDrop that further supports drawing ROIs directly within the XTK framework (http://users.loni.ucla.edu/~pipeline/viewer/). A pediatric brain atlas, also built using the XTK visualization platform, further demonstrates the power of this framework (http://fnndsc.github.io/babybrain/, Figure 5). Another notable web based viewer is Papaya (http://github.com/rii-mango/Papaya), based on a similarly functioned Java client (http://en.wikipedia.org/wiki/Mango_(software)).

## Materials and methods

XNATView was designed as a light-weight version of the bundled XNAT image viewer, which is in essence ImageJ (http://rsb.info.nih.gov/ij/index.html). As mentioned above, this current bundled application is Java-based, and we had difficulty on some of our machines installing the proper version of Java and consistently loading the application.

The initial prototype of XNATView was developed using the Adobe Flex framework, but the current implementation is now written in native JavaScript. XNATView uses a Representational State Transfer (REST) (Fielding, 2000) API to query the XNAT database below, capitalizing on the PyXNAT (Schwartz et al., 2012) library. The back-end functionality is written in Python and the user interface is primarily written in jQueryUI (http://jqueryui.com/).

### Leveraging XNAT's rest interface to develop a customizable image management system

One of the most powerful aspects of the XNAT framework is the introduction of a REST-based API, which allows programmatic access to the available imaging data. In developing XNATView, we have exploited this capability to develop our own image viewer and web-based GUI for image navigation. The PESSCARA framework also supports REST-based queries, allowing us to leverage the XNATView architecture and generalize it to produce a more flexible zero footprint image viewer.

While the rich metadata which XNAT provides related to scan times, quality, echo time, etc. is important to be able to access by “power users,” the REST interface allows our lightweight viewer to sit on top and to abstract many details which may be overwhelming for the average user. In this way, XNATView trades some of the functionality for performance by allowing its users to be able to quickly view the imaging data and accompanying metadata while providing basic image processing tools such as contrast and zoom. The average researcher is allowed to tailor his or her interface and expose select data elements to their user base to allow for cleaner image viewing and annotation. The images are also cached, which clears some of the load away from the XNAT back-end.

### PyXNAT

XNATView's back-end functionality is written in Python and communicates with XNAT via PyXNAT (Schwartz et al., 2012). PyXNAT is a Python library that wraps around the RESTful Web Services provided by XNAT and aims to bridge the communication with an XNAT server and provides an object-oriented approach to querying the data. PyXNAT, combined with other scientific libraries available in Python, allows the user to query, change metadata, upload, and download files in a structured and intuitive way. For example, if we want to get a list of projects in a given XNAT instance (identified by an instance string, username, and password), we would type the following command:

xnat = Interface(server=instance,
  user=username, password=password,
cachedir = os.path.join(os.path.expanduser
  (' '),'XNATVIEW/.store'))
project_list = xnat.select.projects().get()


The variable project_list will now contain all the names of projects in the specific XNAT instance. PyXNAT preserves the hierarchy and data organization in its API, so if we would want to get a list of subjects in a particular project we would type:

xnat.select.project(project_name).
  subjects().get('label')


Downloading and reading DICOM files is also simplified with PyXNAT. We use the dicom package available in Python to read DICOM tags and PyXNAT API to download the files from the online archive to our systems. The following code downloads the DICOM files associated with a scan and reads the instance number in each slice.


for each_file in scan.resource('DICOM').
  files():
    #download from XNAT in tempDir
    path = os.path.join(tempDir,each_file.
      id())
    each_file.get(path,False)
    #read DICOM tags
    dicomData = dicom.read_file(path)
    tag = str(dicomData.InstanceNumber)


Scan is an object that contains information specific to a particular scan, tempDir is a path on the local system, and dicom is the Python package (import dicom).

## Results

### XNATview organization

**Figure 6:**
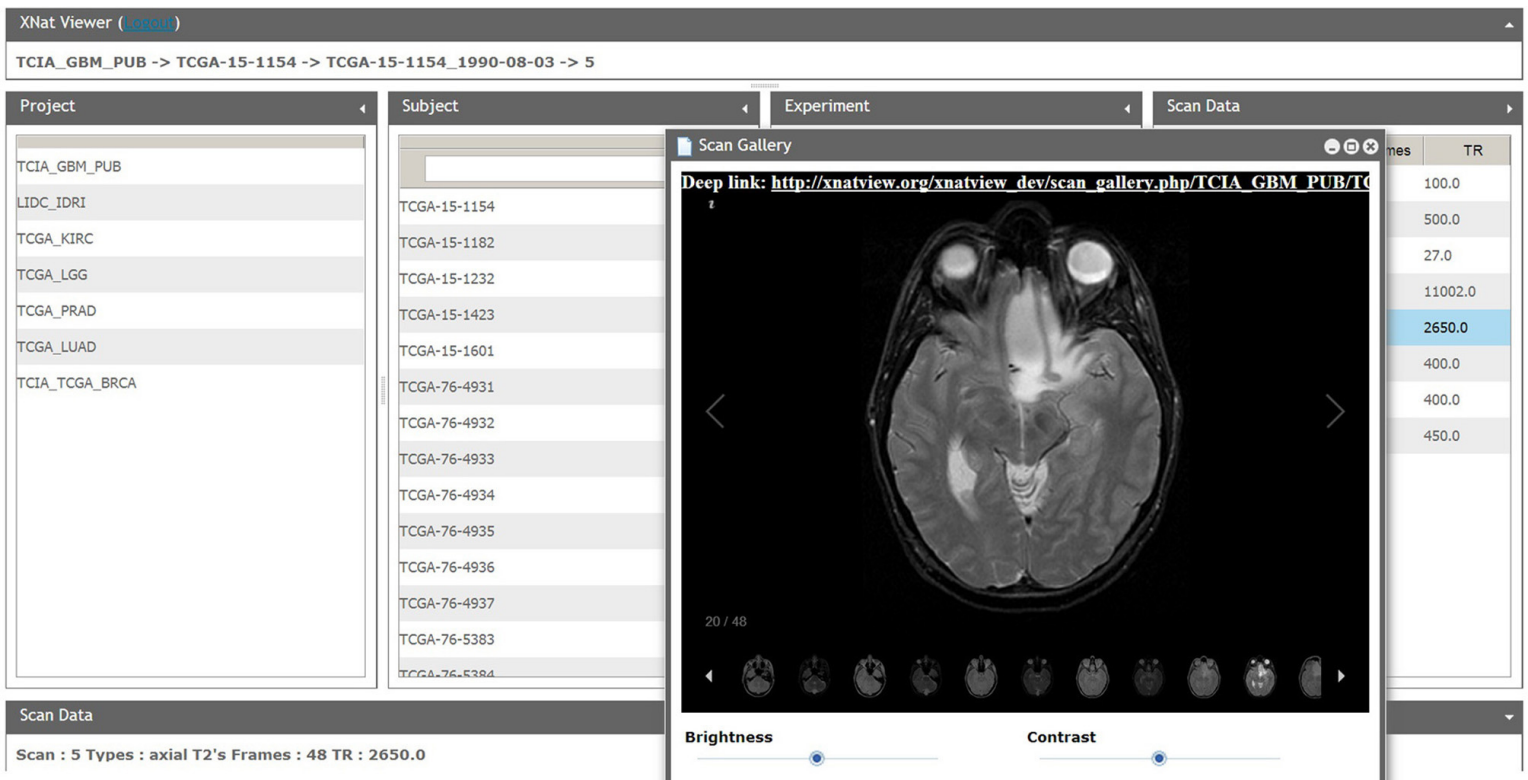
**XNATView user interface**. Users can select a specific scan narrowing search criteria from project, subject, session, and scan from left to right. Selected scans can be custom tiled to suit specific viewing needs, scrolled between slices, and viewed using various image settings such as brightness and contrast.

One of the most important characteristics of XNATView is its very simple and intuitive user interface that allows a user to browse among tens of thousands of images from one centralized location. The data is organized using the same hierarchical concept used in XNAT—the data is grouped left to right according to projects, subjects, experiments, and scan type (Figure 6). The home user interface also allows users to view basic metadata such as patient age and medication regimen associated with the scan as well as easily view, compare, and analyze multiple scans at once.

### Data federation

One interesting aspect of XNATView is the potential to allow multiple systems (XNAT based or other) to present a single federated view of available data sources. Specifically, the ability to exploit the powerful REST interface accessible via PyXNAT allows individual labs to maintain their own imaging repository but share higher level attributes (available subjects, projects, etc.) while still maintaining access to the underlying images.

By facilitating the need to send and manage multiple files for review by collaborators, these interactive tools allow immediate feedback from collaborators. Thus, in addition to improving upon traditional image communication methods involving static images or single slice views, we currently support the communication of images adjusted for brightness/contrast, opacity, color, and other properties of image overlays/masks.

### Enhanced data access

Another valuable feature we have incorporated is the ability to provide a “deep link” to a file. When a user loads an image, a URL appears at the top of the screen; the user can email a colleague the URL and be directly sent to an image of interest. This could be further extended to maintain various desired settings (e.g., contrast, brightness, zoom). As we further integrate the ability to visualize image masks, this feature will become even more useful.

As another example of this flexibility, we have developed a data-finding utility we internally dubbed “XNAT Soup.” As we were trying to group and visualize image series with similar scan parameters, we developed an application that allows us to visualize collection of images with similar scan properties. XNAT Soup includes some standard visualizations, such as scatterplots, but the main feature is a novel visualization for identifying relationships between groups of scans. The visualization uses a force-based layout that supports dragging, panning and zooming. Each subject is represented as a single node. A repulsive force causes subject nodes to spread out so as not to appear on top of one another and a drag force prevents this from causing them to fly out of view.

XNAT Soup utilizes XNAT's REST-based search-engine API to allow the user to execute any scan queries that are supported by XNAT itself. Each search query is added to the visualization as a new node, which we call a *scan group node*. The size of each scan group node is determined by the number of scans included in the group. For each subject node that has scans in the scan group, a spring-force edge is added between the subject node and scan group node. The strength of the spring force is determined by the percent of scans in the scan group that belong to that subject.

**Figure 7:**
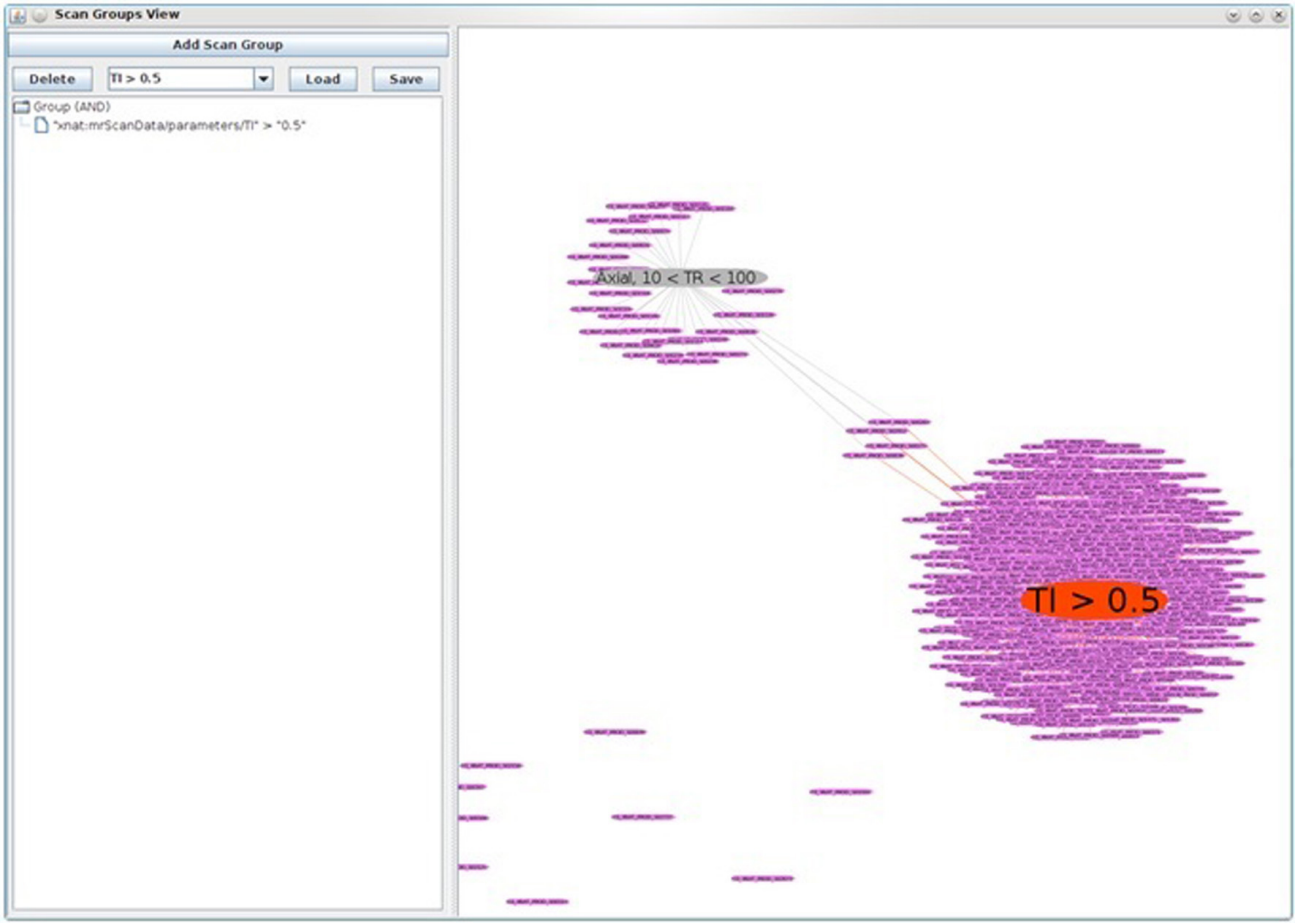
**XNAT Soup, a data finding utility used to visualize collections of images with similar scan properties**. In this figure, two scan groups are shown: (1) the smaller group, in gray, includes only axial scans with a TR value between 10 and 100, while (2) the larger group, in red, includes all scans with a TI value above 0.5. Note also the four subjects in between, who have scans from both groups.

The end result is that subjects related to a scan group are drawn close to it and follow it when the scan group node is moved around. Any node can be double-clicked to force its position to remain stationary. When there are multiple scan groups present, some subjects will appear between the two when they have scans in both groups. This can be used to identify groups of subjects with particular characteristics. Hovering over any node reveals additional details in a tooltip. Figure 7 shows an example where two scan groups are shown: (1) the smaller group, in gray, includes only Axial scans with a TR value between 10 and 100, while (2) the larger group, in red, includes all scans with a TI value above 0.5. From this view, we can see that there are four subjects with scans in both groups.

### Zero-installation feature

Finally, to keep up with rapidly evolving technology, we are quickly moving toward a zero-installation model for MRI visualization and manipulation. This allows for a centralization of image data which helps with the problem of file versioning as the original file is not downloaded but only streamed from the server to the webclient. Although there are pragmatic concerns, such as the ubiquity of outdated Internet Explorer installations on many machines, the technical burden of sharing and interacting with images is rapidly decreasing. This then offers the possibility of interaction and feedback from colleagues with a wide degree of technical expertise and further fosters collaboration and knowledge discovery.

### XNATview operation

XNATView allows the user to choose an XNAT instance and log-in using XNAT credentials for that instance. The flexibility of the REST-based services in XNAT allows the XNATView to run without any modification of XNAT itself, and can communicate with any accessible XNAT back-end. In addition, various visualization plugins and our basic 2-D slice viewer as well as experimental support for an XTK based 3D visualization tool are supported by our XNATView. The basic XNATView, interface supports Internet Explorer 8 with limited functionality, which is often the standard browser on many hospital and clinic settings. A public instance of XNATView is available at http://xnatview.org/ (This version offers basic user options and is available by clicking “guest” at http://xnatview.org/), which mirrors publically available data provided by the Cancer Imaging Archive (Prior et al., 2013).

## Discussion

To support various neuroimaging research demands in our lab, we have developed XNATView, a tool that interfaces with XNAT and leverages the REST layer which XNAT exposes for programmatic data access. As we further develop this, we hope to generalize this software into a Zero Foot Print Image Viewer (ZFIV) which can support multiple back-ends (e.g., not just XNAT or PESSCARA).

XNATView capitalizes on the functionalities of PyXNAT and serves as a lightweight interface that allows easy visualization of a wide range of image series along with metadata. As a result, users are able to review thousands of images from one centralized location, which has the potential to improve data sharing and collaboration (Walden et al., 2011). Some advantages of our implementation are the ability to provide “deep links” allowing users direct access to a particular scan/session, the potential to provide federated views to multiple backends (XNAT or other), a simple UI, Internet Explorer support back to version 8 (with somewhat reduced functionality), and removing the dependency on Java.

It is important to highlight the extreme flexibility that both PyXNAT and XNAT allow via the REST based user interface. REST is a powerful tool used to access, query, retrieve and convert database entries and we chose this platform based on its ease of utilization and functional flexibility. In fact, it has been used in several applications ranging from displaying bioinformatics data from sequence alignment data (Katayama et al., 2010) to assisting physicians in drug prescription decisions (Bianchi et al., 2013).

Therefore, while we feel the application is itself of interest, perhaps the most important aspect of this work is the ability to leverage the power of REST-based mechanisms to allow “mash-ups.” In essence, XNATView is a simple thumbnail gallery, similar to the multitude of image viewers available. The ability to expose data via REST, however, allows the end-user to repurpose and abstract many of the functions of the underlying tool (XNAT) to suit their own needs. As discussed above, our current work with PESSCARA, which also supports REST based image query and retrieval, can be similarly attached. Of note, the name XNATView reflects the initial implementation of this framework, although as we enable other back-ends, ZFIV may be a more appropriate moniker.

## Code availability

The code and initial application is available at our github site [https://github.com/dgutman/ZeroFootPrintImageViewer_XnatView].

## Funding information

This work was supported in part by PHS Grant UL 1RR025008 from the Clinical and Translational Science Award Program, National Institutes of Health, Contract No HHSN261200800001E from the national Cancer Institute, National Institutes of Health, and the Georgia Research Alliance.

### Conflict of interest statement

The authors declare that the research was conducted in the absence of any commercial or financial relationships that could be construed as a potential conflict of interest.
